# The complete chloroplast genome sequence of *Camellia zhaiana* (Theaceae), a critically endangered species from China

**DOI:** 10.1080/23802359.2021.1955027

**Published:** 2021-08-01

**Authors:** Fang-Yuan Wu, Shi-Cheng Ma, Pin-Ming Ye, Hang Ye, Jin-Lin Ma

**Affiliations:** a Guangxi Key Laboratory of Special Non-wood Forest Cultivation and Utilization, Guangxi Forestry Research Institute, Nanning, China; b Wuzhou Tea Industry Development Service Center, Wuzhou, China; c Golden Camellia Park, Nanning, China

**Keywords:** *Camellia zhaiana*, chloroplast genome, Illumina sequencing, phylogenetic analysis

## Abstract

*Camellia zhaiana* S.X. Yang (Theaceae) is a recently described species reported from Guangxi, China. It was proposed as a critically endangered species according to the IUCN Red List Categories and Criteria. In this study, we report and characterize the complete chloroplast (cp) genome of *C. zhaiana* using Illumina pair-end sequencing data. This is the first report of a cp genome of a species classified in *Camellia* section. *Longipedicellata*. The cp genome of *C. zhaiana* is 156,627 bp in length and includes a large single-copy region (LSC, 86,196 bp), a small single-copy region (SSC, 18,281 bp), and a pair of inverted repeat regions (IRs, 26,075 bp). The genome contains 135 genes, including 40 tRNA, eight rRNA, and 87 protein-coding genes. Phylogenetic analysis showed a strongly supported sister relationship between *C. zhaiana* and *C. longipedicellata*, which is a species classified in sect. *Longipedicellata.* These data support the previous systematic findings of *C. zhaiana* and advance the bioinformatics of the genus *Camellia*.

*Camellia* contains about 120 species distributed in East and Southeast Asia (Ming 2000; Ming and Bartholomew 2007). The Southern Yangtze River of China is the center of species diversity for the genus (Ming and Zhang 1996). *Camellia zhaiana* is one of these species and was recently reported as new to science (Liu et al. 2020). It was said to be classified in sect. *Longipedicellata*, and to resemble *C*. *longipedicellata* (Liu et al. 2020). It grows in the evergreen broad-leaved forests of limestone mountains at the elevation range 30-100 m (Liu et al. 2020). It is distinct in being the only species from China with red flowers and a long pedicle (Liu et al. 2020). Although the morpho-anatomy of *C. zhaiana* was well described, no DNA sequences of this species are published. In this study, we present the complete chloroplast (cp) genome sequence of *C. zhaiana* to determine its systematic position in *Camellia* and to contribute to the future phylogenetic and taxonomic studies of the genus.

Fresh leaves of *C. zhaiana* were collected from Long’an county of Guangxi, China (23°05′7.00″N, 107°44′2.91″E). The voucher specimen (S.X. Yang, P. M. Ye & F. Y. Wu 6023) was deposited at the Herbarium at Kunming Institute of Botany (KUN, http://www.kun.ac.cn, Jing-Hua Wang, wangjh@mail.kib.ac.cn), Chinese Academy of Sciences. Total genomic DNA was extracted using a modified hexadecyltrimethylammonium bromide (CTAB) method (Doyle and Doyle 1987). The 150 bp pair-end reads were generated using the Illumina Hi-Seq 2500 platform. The clean data was de novo assembled by GetOrganelle (Jin et al. 2020), followed by using Bandage 0.8.1 (Wick et al. 2015) to assess the completeness of the assembly. PGA (Qu et al. 2019) was used to annotate the chloroplast genome using the default settings. Phylogenetic analysis of *C. zhaiana* was performed with 26 *Camellia* species and two outgroups (*Polyspora axillaris* and *Pyrenaria oblongicarpa*) using RAxML version 8.2.12 (Stamatakis 2014) with the GTR + GAMMA nucleotide substitution model and 1,000 bootstrap replicates following a previous study (Yu et al. 2017).

The circular complete chloroplast genome of *C. zhaiana* (GenBank accession number is MW755302) is 156,627 bp in length, with a mean sequencing depth of 70.6×. The GC content of the genome is 37.3%. This genome includes a large single-copy region (LSC, 86,196 bp), a small single-copy region (SSC, 18,281 bp), and two inverted repeat regions (IR, 26,075 bp). In total, it contains 135 genes, with 40 tRNA, eight rRNA, and 87 protein-coding genes.

The maximum likelihood phylogenetic tree revealed that *C. zhaiana* and *C. longipedicellata* formed a monophyletic clade with full bootstrap support (BS = 100%) (Figure 1). This result supported the taxonomic treatment that *C. zhaiana* belongs in sect. *Longipedicellata* and resembles *C. longipedicellata* (Liu et al. 2020). According to the classification of Ming (2000), five species were recognized. The cp genome provides a reference for further study on the phylogeny of *Camellia*, as well as the protection and utilization of *C. zhaiana*.

**Figure 1.**
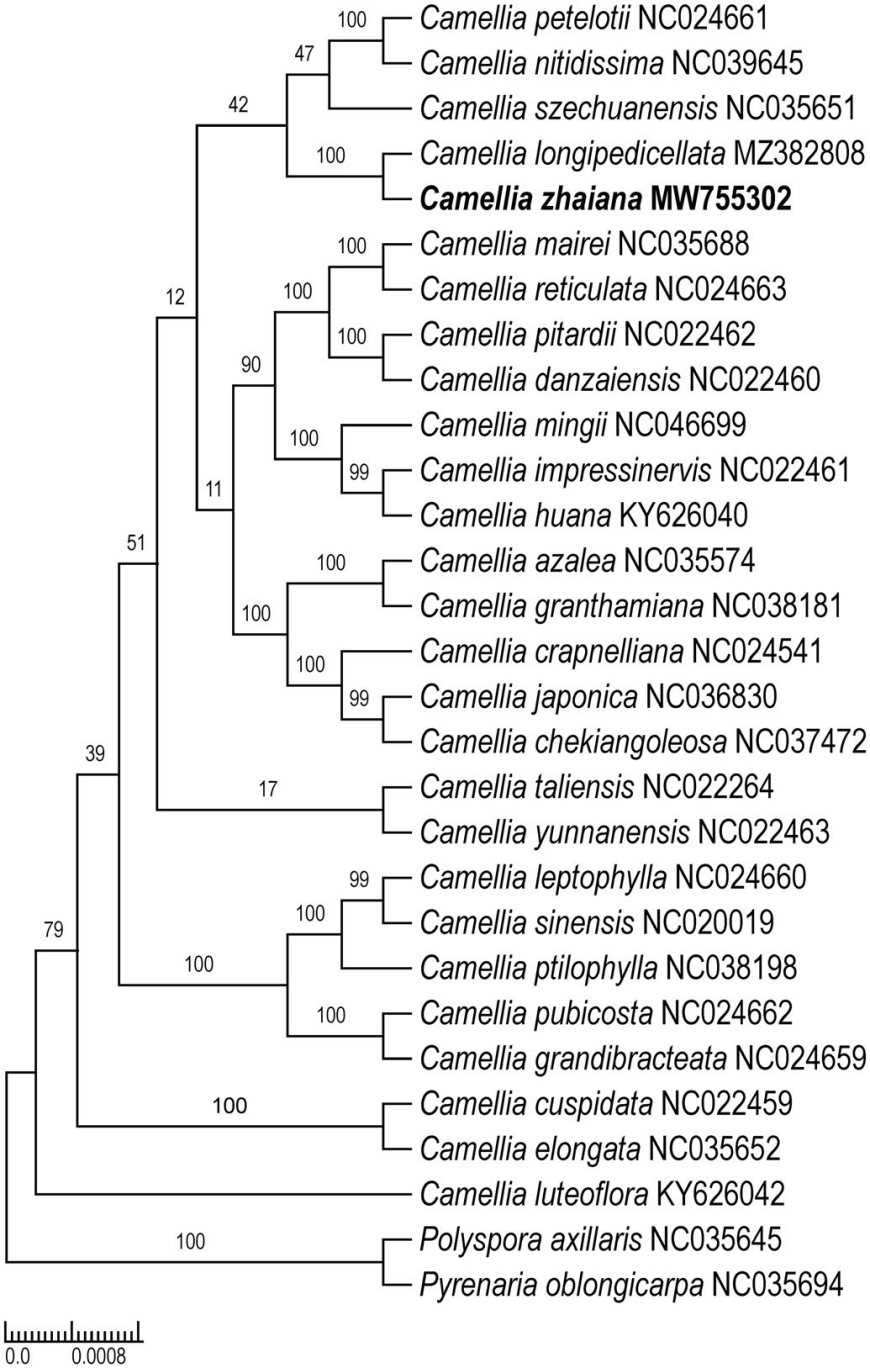
Maximum likelihood tree of Theaceae based on 29 complete chloroplast genome sequences, including *Camellia zhaiana* (GenBank ID: MW755302) sequenced in this study. The bootstrap support values are shown beside the nodes. Two representative taxa of Theaceae (*Polyspora axillaris*, NC035645; *Pyrenaria oblongicarpa*, NC035694) were used as outgroups.

## Data Availability

The data that support the findings of this study are openly available in GenBank of NCBI at https://www.ncbi.nlm.nih.gov/nuccore/MW755302, reference number MW755302. The associated BioProject, SRA, and Bio-Sample numbers are PRJNA725163, SRR14326647, and SRS8774896 respectively.
